# Multilayer Fluorine‐Free MoBT_x_ MBene with Hydrophilic Structural‐Modulating for the Fabrication of a Low‐Resistance and High‐Resolution Humidity Sensor

**DOI:** 10.1002/advs.202404178

**Published:** 2024-07-01

**Authors:** Yong Liu, Yumiao Tian, Fangmeng Liu, Tianyi Gu, Bin Wang, Junming He, Chen Wang, Xing Meng, Peng Sun, Geyu Lu

**Affiliations:** ^1^ State Key Laboratory of Integrated Optoelectronics College of Electronic Science and Engineering Jilin University 2699 Qianjin Street Changchun 130012 P. R. China; ^2^ Key Laboratory of Physics and Technology for Advanced Batteries (Ministry of Education) College of Physics Jilin University 2699 Qianjin Street Changchun 130012 P. R. China; ^3^ International Center of Future Science Jilin University 2699 Qianjin Street Changchun 130012 P. R. China

**Keywords:** first‐principles calculations, fluorine‐free MBene, humidity sensor, hydrothermal‐assisted HCl etching, MoBT_x_, multilayer hydrophilic structure

## Abstract

2D transition metal borides (MBenes) with abundant surface terminals hold great promise in molecular sensing applications. However, MBenes from etching with fluorine‐containing reagents present inert ‐fluorine groups on the surface, which hinders their sensing capability. Herein, the multilayer fluorine‐free MoBT_x_ MBene (where T_x_ represents O, OH, and Cl) with hydrophilic structure is prepared by a hydrothermal‐assisted hydrochloric acid etching strategy based on guidance from the first‐principle calculations. Significantly, the fluorine‐free MoBT_x_‐based humidity sensor is fabricated and demonstrates low resistance and excellent humidity performance, achieving a response of 90% to 98%RH and a high resolution of 1%RH at room temperature. By combining the experimental results with the first‐principles calculations, the interactions between MoBT_x_ and H_2_O, including the adsorption and intercalation of H_2_O, are understood first in depth. Finally, the portable humidity early warning system for real‐time monitoring and early warning of infant enuresis and back sweating illustrates its potential for humidity sensing applications. This work not only provides guidance for preparation of fluorine‐free MBenes, but also contributes to advancing their exploration in sensing applications.

## Graphical abstract

A simpler and safer hydrothermal‐assisted HCl etching strategy is utilized to successfully prepare multilayer fluorine‐free hydrophilic MoBT_x_ MBene. The multilayer hydrophilic structure with oxygen functional terminals is verified by first‐principles calculations and experimental characterization. It is demonstrated that surface physical adsorption and physical intercalation of water molecules are the main mechanisms of MoBT_x_‐based humidity sensors.

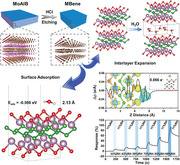

## 1 Introduction

As an emerging group of 2D material, 2D transition metal borides (MBenes) have garnered considerable attention due to their excellent properties, including high electrical conductivity and abundant surface terminals, which make them promising for applications in various fields such as energy storage,^[^ ^1^ ^]^ catalysis,^[^ ^2^ ^]^ medicine,^[^ ^3^ ^]^ magnetic device,^[^ ^4^ ^]^ sensing,^[^ ^5^ ^]^ etc. Especially in the field of molecular sensing, multilayer MBenes with a large specific surface area provide abundant molecular adsorption sites, showing great potential. Furthermore, the surfaces of MBenes are terminated by ─O, ─OH, and other functional groups.^[^ ^6^ ^]^ Among them, the active ─O and ─OH functional groups make materials surfaces more sensitive to water molecules.^[^ ^7^ ^]^ On the other hand, the interaction between MBenes and water molecules may cause changes in the electrical properties of MBenes, which are easily detected and similar to graphene and MXene.^[^ ^7^, ^8^ ^]^ This indicating that their potential as moisture‐sensitive materials for humidity sensing. Furthermore, the high electrical conductivity of MBenes helps reduce power consumption, which is suitable for portable electronics. It is foreseeable that MBenes will attract widespread interest in future sensing research.

The critical factor to be considered in sensing applications is the surface terminals introduced during the preparation process. Multilayer MBenes are typically obtained through the selective etching of the “A” element in the precursor ternary transition metal borides (MAB phases), where M represents the transition metal, B stands for boron, and A denotes the metal layer (III_A_ or IV_A_ element).^[^ ^3^, ^6^ ^]^ MBenes synthesis poses great challenges compared to MXenes due to the presence of different stoichiometric ratios, multiple crystal arrangements, and polymorphism.^[^ ^6b,c^ ^]^ Typically, multilayer MBenes are obtained through etching using fluorine‐containing reagents such as HF and a mixture of HCl/LiF.^[^ ^9^ ^]^ However, the content of surface inert ─F functional groups in MBenes prepared using this type of method can account for a large proportion, which severely affected their physicochemical properties, such as decreased conductivity and hydrophilicity, and ultimately hinders their sensing capabilities.^[^ ^10^ ^]^ Furthermore, the HF is a hazardous poison, which may cause systemic toxicity and fatality in humans.^[^ ^10b^ ^]^ To avoid the use of F‐containing reagents, researchers have employed a high‐temperature dealloying process and a Lewis acid molten salt method to prepare fluorine‐free MBenes, respectively.^[^ ^11^ ^]^ However, these approaches involved more complex preparation processes, even facing the difficulties in synthesizing the corresponding MAB‐phase precursors.^[^ ^11^ ^]^ Therefore, there is an urgent need to develop or improve a simpler and safer fluoride‐free preparation method. Inspired by the strong binding capacity of chloride ions (Cl^−^) and element aluminum (Al),^[^ ^12^ ^]^ MAB‐phases materials with Al layers could theoretically be etched using a single hydrochloric acid (HCl) solution to form soluble AlCl_3_, thus avoid the use of hazardous F‐containing reagents. Additionally, increasing the temperature of the reaction enhances the corrosiveness of HCl, thereby enhancing the etching effect. Consequently, a simple hydrothermal method is considered to assist HCl etching.^[^ ^10^, ^12^ ^]^ As a result, a hydrothermal‐assisted HCl etching strategy could theoretically be utilized to prepare fluorine‐free MBenes from MAB‐phase precursors.

In this work, a milder hydrothermal‐assisted HCl etching method for fluorine‐free MoBT_x_ MBene (where T_x_ represents O, OH, and Cl) was investigated, along with its typical application in the field of humidity sensors. With guidance from first‐principles calculations, fluorine‐free MoBT_x_ with a multilayer hydrophilic structure was successfully obtained from accessible MoAlB precursors by regulating the hydrothermal temperature. The resulting fluorine‐free MoBT_x_ exhibited excellent hydrophilicity and high electrical conductivity. Consequently, it was used as a moisture‐sensitive material to develop a resistive humidity sensor. The results demonstrated that the MoBT_x_‐based humidity sensor exhibited low resistance and excellent humidity performance, achieving a response of 90% to 98%RH and a high resolution of 1%RH. Additionally, assisted by first‐principles calculations and first‐principles molecular dynamics simulation, the humidity sensitivity mechanism, i.e., the interaction between MoBT_x_ and water molecules was revealed for the first time. Furthermore, the portable humidity early warning system enabled real‐time monitoring and rapid warning of enuresis and back sweating in sleeping infants at room temperature, showcasing the potential of MoBT_x_ in humidity sensing applications. To the best of our knowledge, this is the first instance of MBenes materials being utilized in the field of humidity sensors, which will contribute to advancing the development of MBenes materials and their exploration in sensor application.

## 2 Results and Discussion

### 2.1 First‐Principles Prediction of Etching Feasibility via HCl

The etching feasibility of HCl was confirmed through first‐principles calculations. We evaluated the chemical potential of element Al, in MoAlB (µ_*Al*_(*MoAlB*)) and AlCl_3_ (µ_*Al*_(*AlCl*_3_)) since etching fundamentally involves the removal of aluminum atoms.^[^ ^12a^ ^]^ The result revealed that µ_*Al*_(*AlCl*_3_) is −3.49 eV, lower than µ_*Al*_(*MoAlB*) (−3.24 eV). This indicates that Al atoms in the MoAlB phase are more inclined to react and form soluble AlCl_3_ in the presence of Cl^−^ ions. On the other hand, following prior research,^[^ ^9a^ ^]^ and taking into account the possible introduction of different functional group terminals, we can describe the potential reactions occurring during the etching process with the following chemical reaction equations:

(1) $$MoAlB+3HCl=MoB+AlCl_{3}+\frac{3}{2}H_{2}$$

(2) $$MoAlB+3HCl+H_{2}O=MoBO+AlCl_{3}+\frac{5}{2}H_{2}$$

(3) $$MoAlB+3HCl+H_{2}O=MoBOH+AlCl_{3}+2H_{2}$$

(4) $$MoAlB+4HCl=MoBCl+AlCl_{3}+2H_{2}$$

The corresponding reaction free energies (Δ*G*) for these reactions are then calculated as follows:

(5) $$\begin{array}{ccc} \Delta \;G_{MoB} & = & G\left(MoB\right)+G\left(AlCl_{3}\right)+\frac{3}{2}G\left(H_{2}\right)-G\left(MoAlB\right)\\ & & -\;3G\left(HCl\right) \end{array}$$

(6) $$\begin{array}{ccc} \Delta \;G_{O} & = & G\left(MoBO\right)+G\left(AlCl_{3}\right)+\frac{5}{2}G\left(H_{2}\right)-G\left(MoAlB\right)\\ & & -\;3G\left(HCl\right)-G\left(H_{2}O\right) \end{array}$$

(7) $$\begin{array}{ccc} \Delta \;G_{OH} & = & G\left(MoBOH\right)+G\left(AlCl_{3}\right)+2G\left(H_{2}\right)-G\left(MoAlB\right)\\ & & -\;3G\left(HCl\right)-G\left(H_{2}O\right) \end{array}$$

(8) $$\begin{array}{ccc} \Delta \;G_{Cl} & = & G\left(MoBCl\right)+G\left(AlCl_{3}\right)+2G\left(H_{2}\right)-G\left(MoAlB\right)\\ & & -\;4G\left(HCl\right) \end{array}$$

where G is the Gibbs free energy. Based on first‐principles calculations, the obtained values for Δ*G_MoB_*, Δ*G_O_*, Δ*G_OH_* and Δ*G_Cl_* are −1.225, −2.525, −2.147, and −2.873 eV, respectively. These negative values indicate the probability likelihood of the corresponding reactions occurring, with smaller values suggesting more favorable reaction pathways. These comparative results further support the feasibility of using HCl to etch MoAlB, indicating a tendency to produce functionalized MBene, i.e., MoBT_x_ (where T_x_ represents O, OH, and Cl).

### 2.2 Experimental Preparation and Characterizations of MoBT_x_

**Figure 1:**
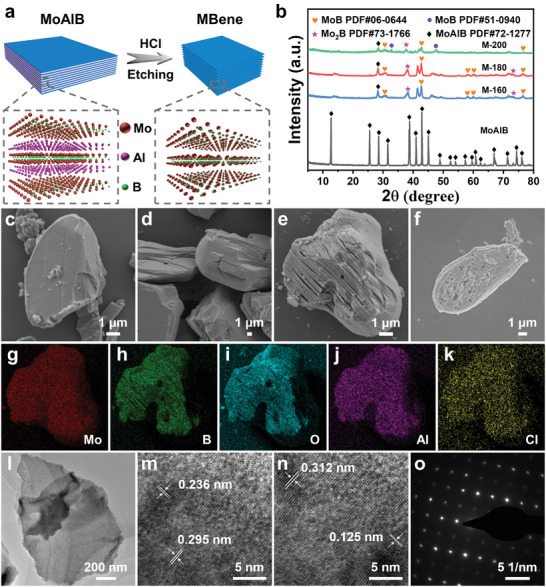
a) Schematic illustration for the preparation of MBene. b) The XRD patterns of MoAlB, M‐160, M‐180 and M‐200. SEM images of c) MoAlB, d) M‐160, e) M‐180 and f) M‐200. g–k) EDS element mapping images of MoBT_x_. l) TEM image of MoBT_x_. m,n) HRTEM images of MoBT_x_. o) A SAED pattern of MoBT_x_.

Based on the calculated results, a strategy for HCl etching with hydrothermal assistance was developed, and the effect of reaction temperature was examined via characterization analysis. In the experiments, hydrothermal temperatures of 160, 180, and 200 °C were employed, yielding products denoted as M‐160, M‐180, and M‐200, respectively, as illustrated in **Figure** **1**a. To investigate the composition and morphological structure of the etched products, various characterization methods were used. Figure 1b illustrated the x‐ray diffraction (XRD) patterns of MoAlB, M‐160, M‐180, and M‐200. The diffraction peaks observed in the MoAlB precursor powder were basically consistent with those listed in the standard card PDF#72‐1277. Post‐etching, new diffraction peaks appeared, with those peaks of M‐160 and M‐180 potentially corresponding to MoB (PDF#06‐0644) and Mo_2_B (PDF#73‐1766), respectively. Meanwhile, for M‐200, some emerging diffraction peaks were attributed to MoB (PDF#51‐0940). Notably, the appearance of a diffraction peak at 14.0° post‐etching is obtained from the offset of the (020) peak of MoAlB, indicating the successful transformation of the MoAlB phase into 2D MBene. Unlike typical MXenes, the rightward shift of the (020) peak suggested a reduction in the interlayer spacing of MoAlB after etching, due to the attraction between the two Mo atomic layers, which occur as a result of etching the Al layer. Furthermore, the persistence of some characteristic peaks of MoAlB post‐etching indicated incomplete etching of the material. Additionally, the presence of broad peaks suggested the existence of high density laminar dislocations resulting from the deintercalation of Al from MoAlB.^[^ ^13^ ^]^ Based on the XRD results, it was concluded that MoAlB can effectively etched by HCl with hydrothermal assistance.

The morphology of materials was further characterized using scanning electron microscopy (SEM) and transmission electron microscopy (TEM). Figure 1c–f illustrates the morphology of MoAlB, M‐160, M‐180, and M‐200 using SEM, respectively. The MoAlB precursor powder exhibited a massive structure, while M‐160 had slight layer structure on the surface. Significantly, M‐180 showed a clear and typical multilayer structure after HCl etching. However, the surface of M‐200 appeared heavily corroded, which could be attributed to the increased corrosiveness of HCl at higher temperature.^[^ ^12a^ ^]^ Based on the above results, the optimal product was obtained at 180 °C and the multilayer structure was modulated by hydrothermal temperature regulation. Consequently, M‐180 was selected as the preferred material for all subsequent experiments, unless otherwise stated. The energy dispersive spectrometer (EDS) element mapping of MoAlB (Figure S1, Supporting Information) and MoBT_x_ (Figure 1g–k), confirmed the reduction in Al elemental content and the introduction of oxygen (O) and slight amount of chlorine (Cl) in MoBT_x_ compared to MoAlB. Furthermore, the presence of a small amount of Al atoms in MoBT_x_ had a stabilizing effect on its multilayer structure.^[^ ^14^ ^]^ The TEM analysis revealed the presence of single‐layer or few‐layers MoBT_x_ structures with a size of ≈900 nm (Figure 1l), indicating the successful formation of partial single‐layer (or few‐layers) construction in the obtained MoBT_x_ after sonicated using a column sonicator. Furthermore, high‐resolution TEM (HRTEM) images in Figure 1m,n displayed distinct lattice fringes. The observed lattice fringes with spacings of ≈0.236, 0.295, and 0.125 nm corresponded to the (002) facet of Mo_2_B (PDF#73‐1766), (110) and (042) crystalline planes of MoB (PDF#06‐0644), respectively. The lattice stripes with a spacing of ≈0.312 nm were attributed to the (110) face of MoAlB (PDF#72‐1277). It can be clearly observed from Figure 1o that the selected region was a typical single crystal structure belonging to the orthogonal crystal system, which is not commonly observable in MXenes. This indicated the presence of MoB, Mo_2_B and some unetched MoAlB in the MoBT_x_, which is consistent with the XRD results. The SEM and TEM results visually demonstrated the effective etching of MoAlB, yielding MoBT_x_ with a distinctive multilayer structure at 180 °C.

**Figure 2:**
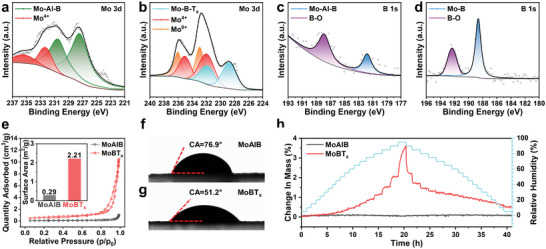
The Mo 3d XPS spectra of a) MoAlB and b) MoBT_x_. The B 1s XPS spectra of c) MoAlB and d) MoBT_x_. e) Nitrogen adsorption‐desorption isotherms of MoAlB and MoBT_x_. The water contact angle (CA) of f) MoAlB and g) MoBT_x_. h) The DVS curves of MoAlB and MoBT_x_ at room temperature.

The changes in elemental valence, specific surface area, and hydrophilicity before and after etching were investigated using x‐ray photoelectron spectroscopy (XPS), Brunauer‐Emmett‐Teller analysis, water contact angle and dynamic vapor sorption (DVS) measurements, respectively. From **Figure** **2**a,b, it is evident that two pairs of Mo 3d_5/2_ (227.5 eV, 232.4 eV) and Mo 3d_3/2_ (232.4 eV, 235.5 eV) peaks appeared in MoAlB, corresponding to Mo─Al─B bonding and Mo^4+^, respectively. In contrast, the spectra of the Mo 3d region for MoBT_x_ showed three pairs of peaks (Mo 3d_5/2_ 228.8, 232.0, 233.0, Mo 3d_3/2_ 231.9, 235.1, 236.1 eV), which are attributed to Mo─B─T_x_ bonding, Mo^4+^ and Mo^6+^, respectively. Figure 2c,d illustrates that the characteristic peak of the boron (B) element shifted to a higher binding energy after etching, implying the presence of electronegative O atoms, which is consistent with previous literature reports.^[^ ^9b^ ^]^ Figure S4 (Supporting Information) demonstrates the O 1s high‐resolution spectrum of MoBT_x_. The five diffraction peaks correspond to Mo oxides, MoBO_x_, MoB(OH)_x_, B─O, and O_C_, respectively, where Mo oxide comes from surface oxidation, and O_C_ refers to oxygen adsorbed on the surface, such as water molecules and carbon dioxide. This further confirms the introduction of ─O and ─OH. Furthermore, in Figure 2e, no adsorption saturation occurred at high relative pressure and the N_2_ adsorption/desorption isotherm of MoBT_x_ exhibited type IV isotherms with H3 hysteresis loops,^[^ ^15^ ^]^ indicating a layered structure of MoBT_x_. Compared to MoAlB, the specific surface areas of MoBT_x_ increased by seven times, as shown in the inset of Figure 2e, which facilitates the adsorption of water molecules. Additionally, Figure 2f,g exhibits the results of water contact angle tests for MoAlB and MoBT_x_. The water contact angle of MoBT_x_ decreased compared to the precursor, indicating an enhanced surface hydrophilicity resulting from the introduction of terminal functional groups.^[^ ^6^, ^16^ ^]^ In Figure 2h, the mass of MoAlB was almost constant, while the mass of MoBT_x_ gradually increased as relative humidity rises. This indicates that the hygroscopicity of MoBT_x_ is much stronger than MoAlB, which may be attributed to the multilayer structure providing more adsorption sites as well as hydrophilic terminal functional groups on the surface. These observations collectively suggest that the HCl etching was effective, yielding MoBT_x_ with stronger hydrophilicity.

### 2.3 Humidity Sensing Performances of the MoBT_x_ Sensor

**Figure 3:**
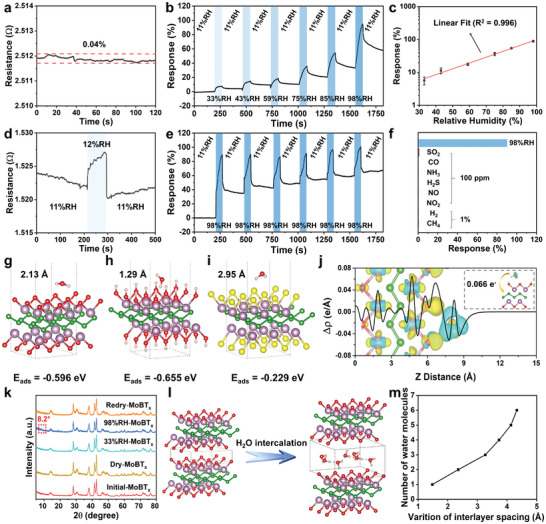
a) The real‐time resistance and the noise level of MoBT_x_ sensor at 11%RH. b) The response curves of the MoBT_x_ sensor to different relative humidity at room temperature. c) The dependence between the logarithm of the response and the relative humidity at room temperature. d) Response curve of the MoBT_x_ sensor to 1%RH change. e) The response curves of the MoBT_x_ sensor to 98%RH for six consecutive times at room temperature. f) The responses of the MoBT_x_ sensor to various gases at room temperature. Water molecules adsorbed on the surface of g) MoBO, h) MoBOH and i) MoBCl. Color code: Mo, purple; B, green; O, red; H, grey and Cl, yellow. j) The plane‐averaged charge density difference for MoBO under H_2_O adsorption. k) XRD of MoBT_x_ stored for 24 h under different conditions. l) Schematic representation of interlayer expansion due to H_2_O intercalation. m) Relationship between the variation of interlayer spacing and the number of intercalated water molecules.

Based on previous findings, MoBT_x_, has shown strong hydrophilicity, making it a promising material for humidity sensors. To investigate the moisture‐sensitive performance of MoBT_x_‐based sensors, we fabricated humidity sensors by applying drops of MoBT_x_‐containing dispersions on interdigital electrodes (Figure S7, Supporting Information). The MoAlB device, fabricated using the same procedures, exhibited a resistance beyond the measurable range of the multimeter (1000 MΩ), as depicted in Figure S8 (Supporting Information), indicating the challenge of further exploring the MoAlB‐based sensor at room temperature. Differently, the MoBT_x_‐based sensor exhibited a significantly lower resistance of only a few ohms of at room temperature (**Figure** **3**a), suggesting high electrical conductivity of MoBT_x_. Furthermore, the power consumption of the sensor was calculated to be ≈2.2 µW (Table S1, Supporting Information). This indicates that the MoBT_x_‐based sensor could operate at room temperature with ultra‐low power consumption for energy savings. The significant change in resistance also suggests the effective etching of MoAlB. The high electrical conductivity of MoBT_x_ arises from the overlap of conduction and valence bands resulting from layer‐to‐layer interactions.^[^ ^17^ ^]^ The resistance the MoBT_x_‐based sensor showed minimal variation (0.04%) at 11%RH, indicating a high signal‐to‐noise ratio for real‐time monitoring, as illustrated in Figure 3a. The responses of the MoBT_x_‐based sensor to different concentrations of H_2_O showed enhancement with increasing RH, featuring a wide detection range (11%RH‐98%RH), as shown in Figure 3b. The increase in H_2_O adsorbed on MoBT_x_ with increasing relative humidity resulted in a weakened electrical conductivity of the MoBT_x_, which is similar to graphene and MXene.^[^ ^7^, ^18^ ^]^ Figure 3c demonstrates a linear correlation (R^2^ = 0.996) between the logarithm of the response value and the relative humidity at room temperature. The response increases more significantly as humidity rises. The MoBT_x_‐based sensor shows good response‐recovery characteristics to changes as small as 1%RH (Figure 3d), highlighting its high detection resolution. **Table** **1** compares the results of this work with other humidity sensors in terms of resistance magnitude and humidity resolution, indicating that the MoBT_x_‐based sensor has extremely low resistance and high detection resolution. The response curves maintain excellent consistency over six consecutive cycles (Figure 3e). The six responses of the sensor to 98%RH are ≈90%, indicating good short‐term repeatability. To investigate the interference in humidity sensing, the MoBT_x_‐based sensor was exposed to 11% RH along with several common gases (Figure 3f). The sensor exhibits a superb selectivity to all the interferential gases, revealing its significant potential for application in complex environments requiring humidity monitoring. As can be observed from Figure S11 (Supporting Information), the response time for the sensor to reach 90% of the response value is 43 s, but the curve cannot fully recover to the initial state, likely due to strong adsorption of H_2_O on the MoBT_x_ surface. Furthermore, the gradual decay of the MoBT_x_ sensor's response value with time (Figure S12, Supporting Information) and the large hysteresis value of the MoBT_x_ sensor (Figure S13, Supporting Information) are related to the incomplete recovery characteristics of the MoBT_x_ sensor. In short, the MoBT_x_‐based sensor demonstrates low power consumption and good response characteristics to different relative humidity levels.

**Table 1:** Comparison of the current study results with resistance magnitudes and humidity resolution from other resistive humidity sensors in recent years.

Sensing Material	Resistance Magnitude (Ω)	Humidity Resolution (RH)	Ref.
MoBT_x_ MXene/MWCNT MXene/PVA MXene‐based smart fabrics 2D MoS_2_ + PEO rGO/MoS_2_ rGO GO‐Coolmax poly(ether‐ether‐ketone) COF‐5 film Hexagonal‐WO_3_ ZnO Nanosheets La_0.9_Sr_0.1_FeO_3‐δ_ Cs_2_SnCl_6_ Cs_3_Bi_2_Br_9_	×10^0^ ×10^4^ ×10^6^ ×10^2^ ×10^6^ ×10^4^ ×10^5^ ×10^7^ ×10^8^ ×10^11^ ×10^8^ ×10^8^ ×10^9^ ×10^8^ ×10^11^	1% 10% 10% 7% 5% 5% 10% 5% 9% 5% 8% 20% 2% 17% 5%	This work [31] [32] [33] [34] [35] [36] [37] [38] [39] [40] [41] [42] [43] [44]

A comprehensive investigation of the response characteristics of the MoBT_x_‐based sensor necessitates an examination of the adsorption and intercalation of water molecules. In this regard, the adsorption energy of water molecules on the MoBT_x_ surface was calculated. Figure 3g–i determines that H_2_O could adsorb on the MoBO, MoBOH, and MoBCl surfaces with binding energies of −0.596, −0.655, and −0.229 eV, respectively, and at the distances of 2.13, 1.29, and 2.95 Å from the surface, correspondingly. Compared to the typical adsorption energy of gas molecules on the MXene surface (−0.1–0.4 eV),^[^ ^19^ ^]^ MoBO and MoBOH exhibit strong physical adsorption of H_2_O. Besides, based on previous calculations of reaction free energies and EDS characterizations, it is evident that the predominant functional group on the MoBT_x_ surface is oxygen (MoBO). Therefore, the strong physical adsorption capability of MoBT_x_ for H_2_O is attributed to the presence of oxygen functional groups.^[^ ^7a^ ^]^ The electron transfer behavior of H_2_O on the MoBO surface was further investigated through charge density difference and Bader charge analysis. A water molecule transfers 0.066 *e* ^−^ to the MoBO surface with electrons accumulating across multiple atomic layers of MoBO (Figure 3j). As a result, the resistance of MoBT_x_ increased after receiving the electrons from H_2_O. This exhibits p‐type sensing behavior, which is similar to graphene^[^ ^8a^ ^]^ and MXene,^[^ ^7^, ^20^ ^]^ and was successfully verified using the thermoelectric effect (Figure S14, Supporting Information) and Hall effect (Table S2, Supporting Information), respectively. Therefore, the electrons transferred to MoBT_x_ from the physically adsorbed H_2_O were complexed with the holes in the valence band of MoBT_x_, leading to a decrease in the number of charge carriers and an increase in resistance. On the other hand, the intercalation of water molecules into the MoBT_x_ structure was explored. The XRD patterns of MoBT_x_ stored in dry (Dry‐MoBT_x_) and 33%RH (33%RH‐MoBT_x_) environments are essentially identical, as shown in Figure 3k. However, in the XRD pattern of MoBT_x_ stored in a 98%RH environment (98%RH‐MoBT_x_), a small diffraction peak appeared at 8.2°, corresponding to an interlayer spacing of 10.7 Å. Compared with the original layer spacing of 6.3 Å (corresponding to the diffraction peak at 14.0°), the layer spacing increased by 4.4 Å. The disappearance of the 8.2° peak after re‐vacuum drying the 98%RH‐MoBT_x_ at 60 °C for 24 h indicates that the process involved in the emergence of this peak is reversible. It was assumed that the appearance of the peak was due to the expansion of the interlayer spacing caused by the insertion of H_2_O into the MoBO layers. This phenomenon has been observed in research on MXenes.^[^ ^21^ ^]^ To confirm this, the change in interlayer spacing resulting from the insertion of H_2_O into the MoBO layers was simulated (Figure 3l). In the simulation, water molecules were gradually introduced into the interlayer of MoBT_x_, and the system was relaxed after each water molecule to obtain the change in interlayer spacing compared to the initial state. As water molecules were inserted, the interlayer spacing gradually increased and the variation in interlayer spacing eventually converged at ≈4 Å (Figure 3m), which is consistent with the changes observed in the XRD pattern. Additionally, the further decrease in electrical conductivity could be attributed to the electronic decoupling of MoBT_x_ layers caused by the increase in interlayer spacing and charge depletion in the presence of adsorbed H_2_O.^[^ ^8^, ^22^ ^]^ This explains why the logarithm of the response is linearly related to the relative humidity, and the response increases more drastically with increased humidity. Summarizing the above two aspects, the MoBT_x_ humidity sensing mechanism of the sensor is schematically shown in Figure S15 (Supporting Information). Hence, the interactions between H_2_O and MoBT_x_, including H_2_O adsorption and intercalation, are physical processes that result in significant changes in the structural and electrical properties of MoBT_x_.

**Figure 4:**
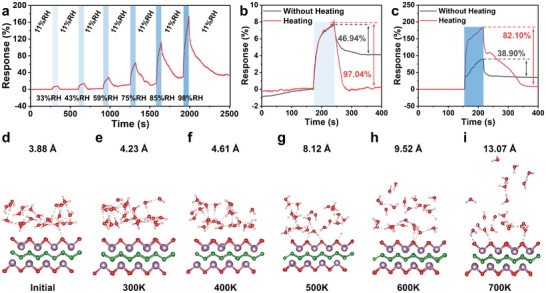
a) The response curves of MoBT_x_ sensor to different relative humidity with heating. Response‐recovery curve of sensor to b) 33%RH and c) 98%RH with heating and without heating. d) The initial structure with water layers. Snapshot of the structure at the end of the 10 ps simulation e) 300K, f) 400K, g) 500K, h) 600K, and i) 700K.

Considering the effect of temperature on the volatilization of water molecules, short‐term heating was utilized during the sensor recovery period to improve the sensor's recovery characteristics. Thermo gravimetric analysis revealed that MoBT_x_ gradually oxidized when the temperature exceeds 203 °C (Figure S17, Supporting Information). Therefore, a heating temperature of 60 °C and a heating time of 60 s were selected for the recovery period of the sensor. The resistance of the sensor under this condition is almost minimally affected by temperature (Figure S18, Supporting Information), indicating that MoBT_x_ is insensitive and stable at short‐term lower temperatures. Compared to the without‐heating condition (Figure 3b), the sensor recovery was substantially improved when heating was applied (**Figure** **4**a), which was due to the thermal detachment of previously confined H_2_O from MoBT_x_ adsorption. Table S3 (Supporting Information) revealed the specific numerical comparison of the recovery level (RL) corresponding to Figures 3b and 4a. Without heating, the RL was only ≈40%, while with temperature‐assisted recovery, the sensor recovered almost completely at low humidity and up to 80% at high humidity. This may be caused by some water molecules entering the interlayer of MoBT_x_ at high humidity. Analogous to MXene with the same structure, the huge lateral size of MXene leads to limited hydration/dehydration of water molecules in the interlayer.^[^ ^19^, ^23^ ^]^ It has been observed that the dynamics of water molecules between the MXene interlayers are much slower compared to water molecules associated with the MXene surface, which is attributed to the fact that the dynamics of the water molecules are affected by van der Waals' forces as they enter the interlayers.^[^ ^8^, ^24^ ^]^ Based on these understandings, it was speculated that H_2_O adsorbed on the surface of MoBT_x_ could almost completely desorb with short‐term heating. However, H_2_O inserted between MoBT_x_ interlayers could still not fully desorb, which led to the recovery level of only 80% at high humidity. Furthermore, as shown in Figure 3k, the disappearance of that peak indicated that water molecules inserted between the MoBT_x_ interlayers could be desorbed by long‐term heating. This suggests that the primary reason for the incomplete recovery of the MoBT_x_ sensor was the strong physical adsorption of water molecules on the MoBT_x_ surface. And the secondary reason was that the dynamics of water molecules are weakened when they entered the MoBT_x_ interlayers at high humidity. To verify the effect of temperature on surface water molecules, a MoBO model with a surface containing a 3.88 Å water layer was used to simulate the short‐term heating of water molecules on the MoBO surface (Figure 4d). Subsequently, ab initio Molecular Dynamics (AIMD) simulations were performed for 10 picoseconds (ps) at different temperatures of 300, 400, 500, 600, and 700 K (Figure 4e–i). The surface‐adsorbed water layer undergone little change at 300 K compared to the initial structure. This minimal change can be attributed to the immobilization of water molecules due to the synergistic effect of hydrogen bonds formed between water layers and the surface. As temperature increased, the surface water layer gradually diffused into the surrounding space owing to the breaking of hydrogen bonds. Furthermore, the diffusion of surface water molecules was found to have a small diffusion barrier, as indicated by the Arrhenius plot (Figure S19, Supporting Information). The diffusion barrier was calculated to be 0.106 eV, suggesting that thermal fluctuations easily surpassed this barrier, enabling the breaking of hydrogen bonds between water molecules and their subsequent diffusion into the surrounding space with increasing temperature. As a result, the recovery level of the MoBT_x_ sensor is increased. On the other hand, the water molecules around the sensor absorb the residual heat from the sensor and their irregular movement is intensified, leading to an increase in the number of H_2_O adsorbed to the sensor in the same amount of time, which ultimately results in an increase in the response of the MoBT_x_ sensor. Accordingly, short‐term heating contributed to the increased recovery level of the sensors, and this was utilized to explore the reasons for incomplete sensor recovery, which are attributed to the strong adsorption of H_2_O on the MoBT_x_ surface and the weakened dynamics of H_2_O in the MoBT_x_ interlayers.

### 2.4 The Portable Humidity Early Warning System

**Figure 5:**
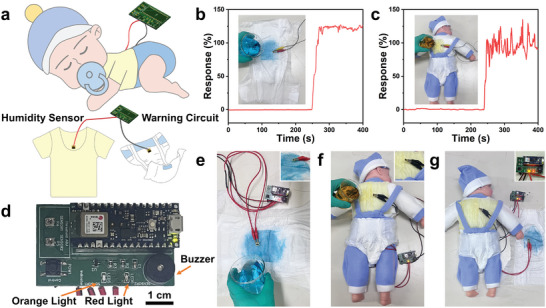
a) Conceptual illustration of humidity sensors for early warning of sleepy babies' enuresis and back sweating. The response curves of the sensor during simulated b) enuresis and c) back sweating at room temperature. d) A physical diagram of the early warning circuit. Early warning state diagrams for simulated e) enuresis, f) back sweating and g) enuresis & back sweating using the portable humidity warning system at room temperature.

To validate the sensor's practicality, we explored its potential for real‐time monitoring of enuresis and back sweating in sleeping infants (**Figure** **5**). Initially, simulated enuresis and back sweating were tested to validate the simulated environments at room temperature, as shown in Figure 5b,c. The response value changed immediately with fluctuations in ambient humidity and stabilized thereafter, meeting the essential criteria for rapid warning. As shown in Figure 5d, the humidity warning circuit was based on the principle that when the sensor encountered changes in ambient humidity causing the resistance to exceeded a specified threshold, the warning light would illuminate and the buzzer would emit a soft beep. Specifically, the red light illuminated for infant enuresis (Figure 5e), the orange light illuminated for back sweats (Figure 5f), and both lights illuminated simultaneously for both enuresis and back sweating (Figure 5g), as demonstrated in the accompanying video (Videos S1 and S2, Supporting Information). Furthermore, the insets of Figure 5e,f display that the sensor was suspended and not in direct contact with the liquid water during the simulation, indicating that the response of sensor was due to the diffusion of water molecules. Overall, the portable humidity early warning system facilitates the monitoring of enuresis and/or back sweating in sleeping infants at room temperature, providing an alert for parents to change diapers or clothes promptly to improve the infants' sleep quality. Additionally, individuals with incontinence, such as those who are paralyzed, demented or are in a vegetative state, can also be benefit from real‐time monitoring using the portable humidity early warning system, as they may have difficulty perceiving or responding promptly to urine loss. This demonstrated the significant potential of the MoBT_x_‐based humidity sensors in daily life.

## 3 Conclusion

In this work, we proposed an effectively strategy for synthesizing fluorine‐free MoBT_x_ MBene through hydrothermal‐assisted HCl etching, guided by first‐principles calculations. The theoretical calculations, considering both chemical potential and reaction free energies, demonstrate the feasibility of HCl etching on MoAlB to yield MoBT_x_ with functional groups. Experimental synthesis confirmed the successful production of fluorine‐free MoBT_x_ with multilayer hydrophilic structures, where oxygen (MoBO) functional terminals predominated on the surface. The effect of hydrothermal temperature on the etching was also investigated. The resulting fluorine‐free MoBT_x_ exhibited desirable properties, including high electrical conductivity and good hydrophilicity. The constructed MoBT_x_‐based sensor demonstrated several favorable characteristics. It exhibited low power (2.2 µW), a high response of 90% to 98%RH, and a wide detection range (11–98%RH) at room temperature. The sensor also exhibited good immunity to interference from other gases and short‐term low temperatures. Assisted by first‐principles calculations and first‐principles molecular dynamics simulation, the humidity sensitivity mechanism of the sensor, i.e., the interactions between MoBT_x_ and water molecules, was revealed for the first time, providing novel insights. It was discovered that water molecules had a strong adsorption energy (−0.596 eV) on the MoBT_x_ surface, hindering desorption and reducing the electrical conductivity of the *p*‐type MoBT_x_ due to electron transfer from H_2_O. As humidity increases, water molecules penetrate the MoBT_x_ interlayers, expending the interlayer spacing by ≈4 Å, further reducing electrical conductivity and leading to significant increase in sensor response with increasing humidity. Moreover, the logarithm of the response was linearly dependent on the relative humidity, indicating a predictable and consistent response. Additionally, the portable humidity early warning system enabled real‐time monitoring and early warning of enuresis and back sweating in infants, establishing their potential as promising sensitive components for sensor applications. In conclusion, this study contributes to the synthesis of fluorine‐free MBenes and explains the interaction of MBenes with water molecules, advancing the development of MBenes materials and validates them as promising sensitive materials for sensors.

## 4 Experimental Section

### Preparation of MoBT_x_ MBene

MoBT_x_ were prepared through a hydrothermal etching method with concentrated HCl. First, 1 g MoAlB (from Jilin 11 Technology Co., LTD.) was slowly added into a 100 mL autoclave with a Teflon liner containing 40 mL 12 m HCl (analytical grade) solution, which was magnetically stirred constantly for 30 min at room temperature. After that, the autoclave was heated to 180 °C (160 or 200 °C) for 24 h in an oven. After cooling to room temperature, the precipitate was washed eight times with deionized water and ethanol alternatively until the pH of the supernatant approached neutrality. The precipitate was then dispersed into deionized water and sonicated using a 300 W column sonicator in an ice bath for 2 h. The dispersion was dried in a vacuum oven at 60 °C for 24 h. Finally, the obtained MoBT_x_ powders were stored in a refrigerator at 4 °C.

### Materials Characterizations

The phase of the products was analyzed by x‐ray diffraction (XRD, Rigaku D/max‐2550 V) with Cu‐Kα radiation (λ = 1.5418 Å) at a scanning speed of 10° min^−1^ over the 2*θ* range of 5°–80°. Scanning electron microscopy (SEM) images and elemental mapping results were obtained using a cold field emission scanning electron microscope (FESEM; JEOL JSM‐7500F) under an accelerating voltage of 5 kV. Transmission electron microscopic (TEM) images and high‐resolution transmission electron microscopy (HRTEM) images were acquired using a TECNAI F20 microscope. The chemical states and compositions were measured using an ESCALAB 250 X‐ray photoelectron spectrometer (XPS) with an X‐ray source (Al Kα hυ = 1486.6 eV). The specific surface area and pore distribution were measured by the Brunauer–Emmett–Teller (BET) equation based on nitrogen adsorption isotherms, employing a Micromeritics Gemini VII instrument (surface area and porosity system). Thermo gravimetric analysis (TGA) was performed using a NETZSCH STA 449F3 thermal analyzer from room temperature to 900 °C under an air atmosphere at a heating rate of 10 °C min^−1^. The water contact angle was measured using a Krüss DSA30 droplet analyzer with a 4 µL water droplet as indicator. The dynamic vapor sorption (DVS) was measured via simultaneous DVS (SPSx‐1µ, Germany)

### Fabrication of Humidity Sensor

The flexible PI substrate (10 × 10 mm) with interdigitated gold/nickel electrodes, each with a width and gaps of the electrodes were 100 nm, was purchased from Huizhou New Wenxiong Trading Co., Ltd. Next, 20 mg of MoBT_x_ powder was dispersed into 1 mL deionized water, sonicated for 2 h and then 20 µL of resulting solution was drop‐casted onto the interdigital electrodes. At last, the device was dried in a vacuum oven at 60 °C for 12 h to eliminate excess moisture.

### Humidity Sensing Characteristics Measurement

The humidity sensing performance was evaluated by a traditional static test method (Figure S9, Supporting Information) at room temperature (23 ± 2 °C), and the real‐time resistance of the sensor was measured with a FLUKE 8846A digital multimeter. Stable ambient relative humidity (RH) of 11%, 33%, 43%, 59%,75%, 85%, and 98% were obtained using the correspondingly supersaturated salt solutions of LiCl, MgCl_2_, K_2_CO_3_, NaBr, NaCl, KCl, and K_2_SO_4_, respectively.^[^ ^25^ ^]^ The resistance of the sensor was measured at different RH (33%, 43%, 59%, 75%, 85%, and 98%), with the resistance at 11% relative humidity as a baseline. 12% RH was obtained by controlling the humidity box (Shanghai ESPC Environmental Equipment Co.). Here, the humidity response is defined as:

(9) $$Response=\left(R-R_{11\%}\right)/R_{11\%}\times 100\%$$

where R is the resistance of the sensor at different RH and R_11%_ represents the resistance of the sensor at 11% RH. The response/recovery time is defined as the time from ΔR (resistance variation) × 0% to ΔR × 90% during response/recovery.^[^ ^7b^ ^]^ During the measurement, the sensor was maintained at 11% RH and different relative humidity levels for ≈3 and 1 min, to ensure uniformity and consistency of the response transients. To provide a more intuitive comparison of the recovery level of the sensor with and without heating, the recovery level (RL) is defined as follows:

(10) $$RL=\left(R_{1}-R_{2}\right)/R_{1}\times 100\%$$

where R_1_ is the response of the sensor at different RH and R_2_ is the stabilized response of the sensor back to 11% RH. For concept demonstration and to enhance the clarity of experimental phenomena, CuSO_4_ and FeCl_3_ solutions were used to simulate urine and sweat, respectively.

First‐Principles Calculations: The Vienna Ab initio simulation package (VASP) was used to perform all of the density functional theory (DFT) calculations.^[^ ^26^ ^]^ The interaction of ion‐electron was described by the projection‐enhanced wave (PAW) method.^[^ ^27^ ^]^ The exchange‐correlation was described using the Perdew–Burke–Ernzerhof (PBE) functional in the generalized gradient approximation (GGA).^[^ ^28^ ^]^ The van der Waals (vdW) interactions present in the multilayer/heterostructure systems were described using the Grimme's DFT‐D3 dispersion correction scheme.^[^ ^29^ ^]^ The cutoff energy of the plane‐wave basis was set to 500 eV. The convergence criteria for force and energy were set to 0.01 eV Å^−1^ and 10^−5^ eV, respectively. A Gamma scheme with a grid density of 0.04 Å^−3^ was adopted. The chemical potential of Al in AlCl_3_ and MAB can be expressed as follows:

(11) $$E\left(AlCl_{3}\right)=\mu_{Al}+3\mu_{Cl}$$

(12) $$E\left(MoAlB\right)=\mu_{Al}+\mu_{B}+\mu_{Mo}$$

where E is energy. The ***µ***_***Mo***_ and ***µ***_***B***_ are referenced to the chemical potential of Mo and B in bulk Mo and borophene. The formula for calculating the Gibbs free energy is

(13) G=E+ZPE−TS

where ZPE represents zero‐point energy, T stands for temperature, and S represents entropy. The adsorption energy of water is calculated as

(14) $$E_{abs}=E_{*H_{2}O}-E_{*}-E_{H_{2}O}$$

where the asterisk (*) represents MoBO, MoBOH or MoBCl.

First‐principles molecular dynamics simulation: Ab‐initio Molecular‐Dynamics (AIMD) simulations of water evaporation on a 4*4 MoBO unit were conducted. The surface contained ≈24 water molecules, roughly two layers. The initial structure was obtained through simulated annealing. Subsequently, AIMD simulations of 10 ps with a 1 fs time step in the canonical ensemble (NVT) were employed to determine the diffusion behaviors of water molecules on the MoBO surface. The diffusion coefficient is defined as^[^ ^30^ ^]^

(15) $$\langle {\left[\vec{r}\left(t\right)\right]}^{2}\rangle =\langle {\left[\vec{r_{0}}\left(t+t_{0}\right)\right]}^{2}-{\left[\vec{r_{i}}\left(t_{0}\right)\right]}^{2}\rangle$$

(16) $$D=\frac{1}{6t}\langle {\left[\vec{r}\left(t\right)\right]}^{2}\rangle$$

where $\vec{r}\left(t\right)$ is the displacement of H_2_O. The relationship between the diffusion barrier (*E_d_*) and the diffusion coefficient (D) is as follows:

(17) $$\log D=\log A-\frac{E_{d}}{k}\times \frac{1}{T}$$

where A is the pre‐exponential factor, k is the Boltzmann constant.

## Conflict of Interest

The authors declare no conflict of interest.

## Supporting information

Supporting Information

Supplemental Video 1

Supplemental Video 2

## Data Availability

The data that support the findings of this study are available from the corresponding author upon reasonable request.
