# Supplementary material for: Multilayer Fluorine‐Free MoBTx MBene with Hydrophilic Structural‐Modulating for the Fabrication of a Low‐Resistance and High‐Resolution Humidity Sensor
Source: Adv Sci (Weinh). 2024 Jul 1;11(33):2404178. doi: 10.1002/advs.202404178 (PMC11434212; doi:10.1002/advs.202404178)
Supplement: Supplementary file 1 — Supporting Information [file ADVS-11-2404178-s002.pdf]

ADVANCED SCIENCE
Open Access

Supporting Information

for *Adv. Sci.*, DOI 10.1002/advs.202404178

Multilayer Fluorine-Free $MoBT_x$ MBene with Hydrophilic Structural-Modulating for the Fabrication of a Low-Resistance and High-Resolution Humidity Sensor

Yong Liu, Yumiao Tian, Fangmeng Liu, Tianyi Gu, Bin Wang, Junming He, Chen Wang, Xing Meng*, Peng Sun* and Geyu Lu*

Supporting Information

Multilayer Fluorine-Free $MoBT_x$ MBene with Hydrophilic Structural-Modulating for the Fabrication of a Low-Resistance and High-Resolution Humidity Sensor

Yong Liu, Yumiao Tian, Fangmeng Liu, Tianyi Gu, Bin Wang, Junming He, Chen Wang, Xing Meng,* Peng Sun,* and Geyu Lu*

Y. Liu, F. Liu, T. Gu, B. Wang, J. He, C. Wang, P. Sun, G. Lu

State Key Laboratory of Integrated Optoelectronics, College of Electronic Science and Engineering, Jilin University, 2699 Qianjin Street, Changchun 130012, P. R. China

*Corresponding author: liufangmeng@jlu.edu.cn, pengsun@jlu.edu.cn

Y. Tian, X. Meng

Key Laboratory of Physics and Technology for Advanced Batteries (Ministry of Education), College of Physics, Jilin University, 2699 Qianjin Street, Changchun 130012, P. R. China

*Corresponding author: mengxing@jlu.edu.cn.

F. Liu, P. Sun, G. Lu

International Center of Future Science, Jilin University, 2699 Qianjin Street, Changchun 130012, P. R. China

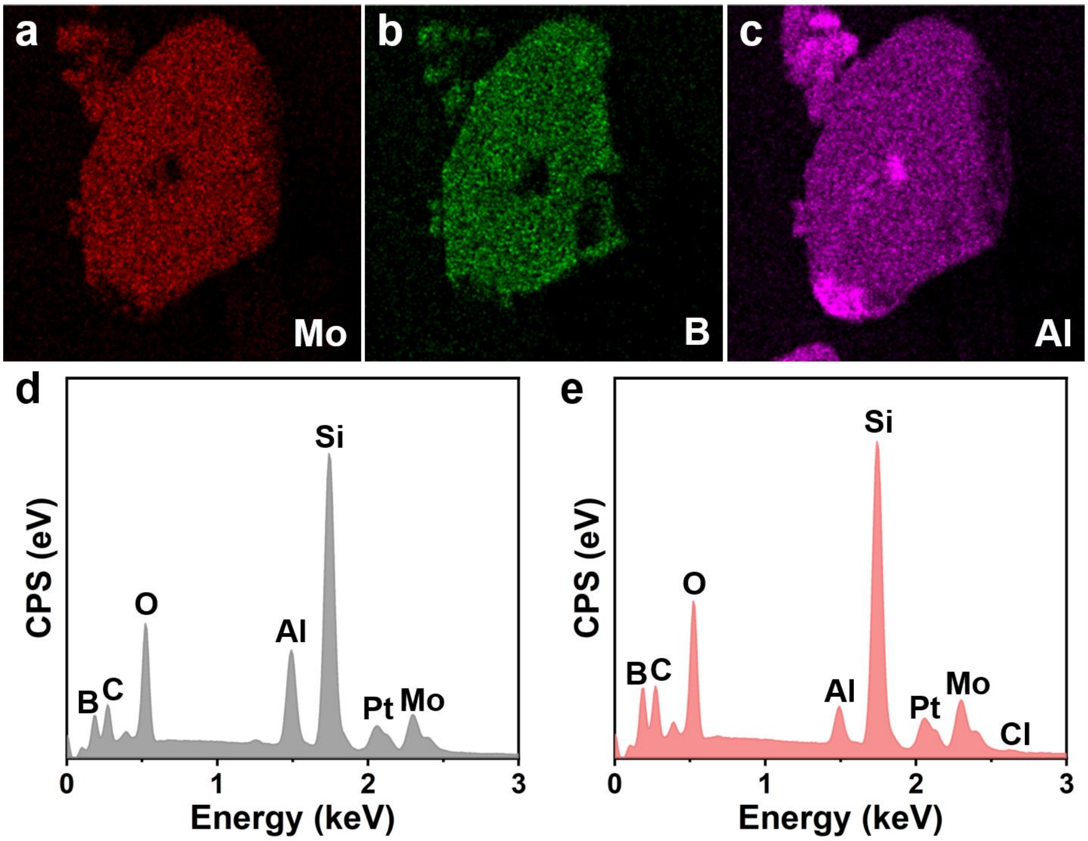

Figure S1. Mapping images of (a-c) MoAlB; SEM coupled with EDS spectra for (d) MoAlB and (e) $MoBT_x$.

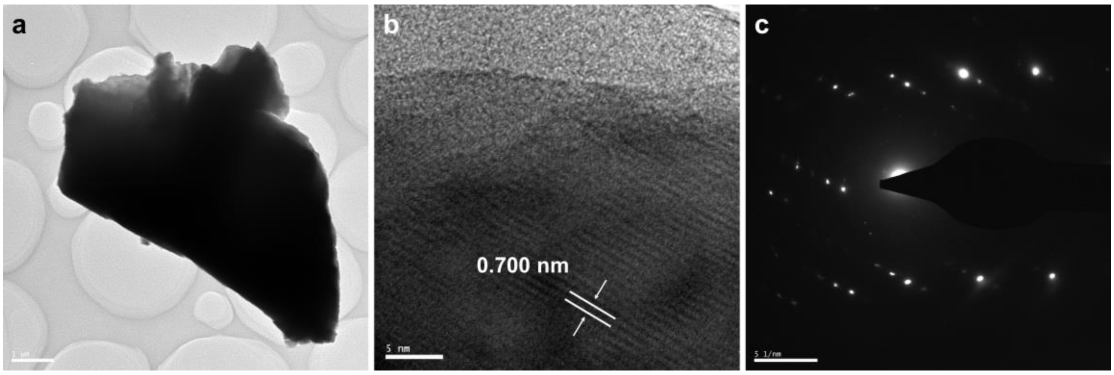

Figure S2. (a) TEM image of MoAlB. (b) HRTEM image of MoAlB. (c) SAED pattern of MoAlB.

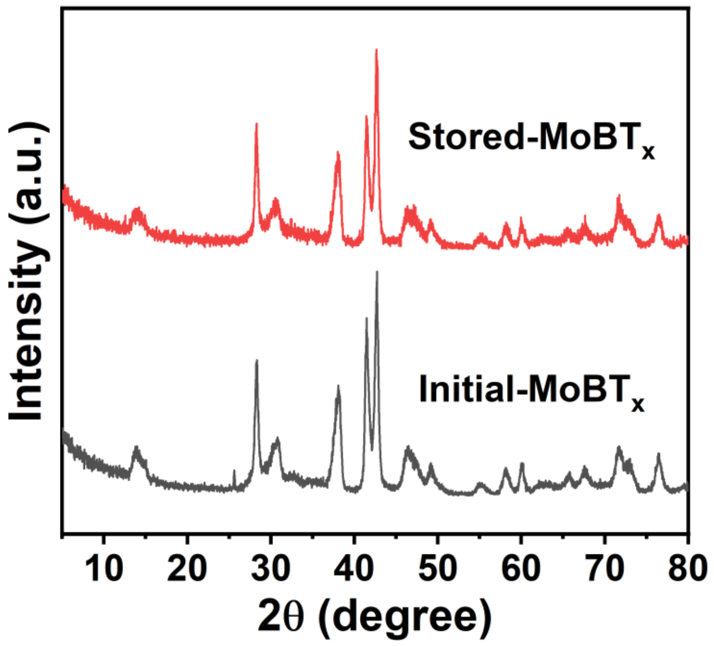

Figure S3. XRD patterns of Initial-$MoBT_x$ (freshly synthesized $MoBT_x$) and Stored-$MoBT_x$ ($MoBT_x$ stored at 4 °C for 14 days).

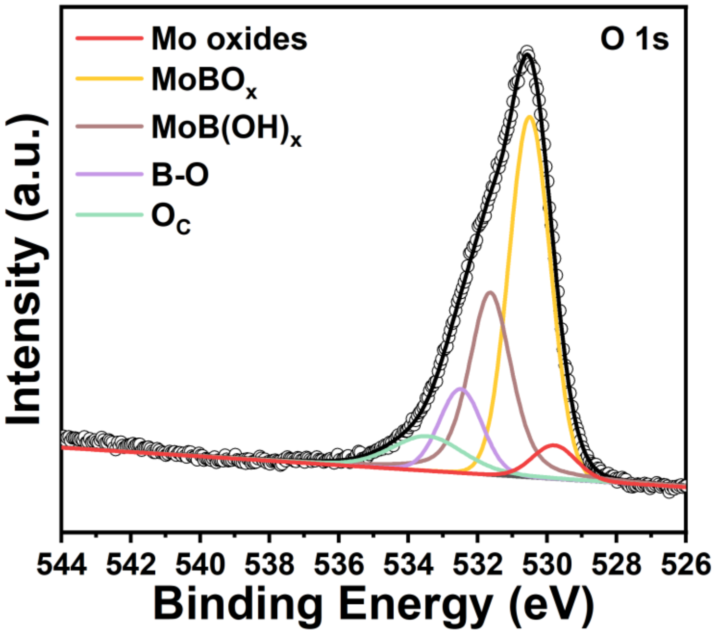

Figure S4. The O 1s XPS spectra of $MoBT_x$.

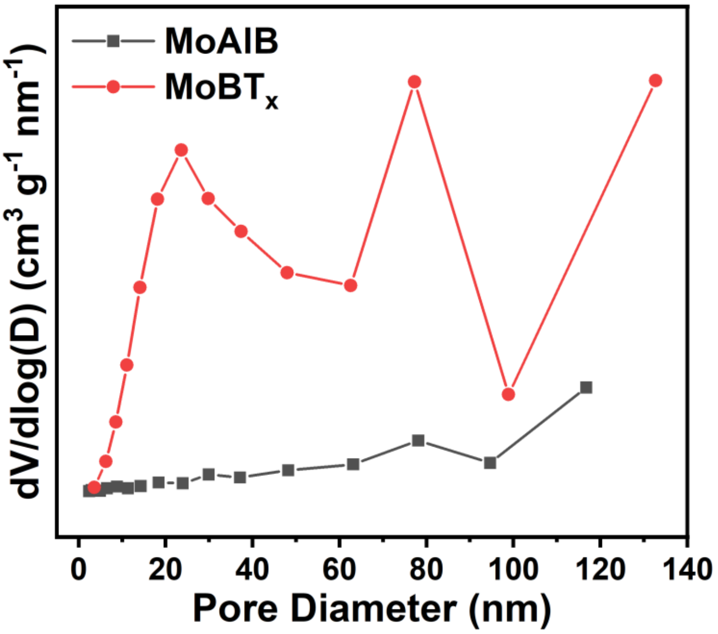

Figure S5. The pore-size distributions of MoAlB and $MoBT_x$.

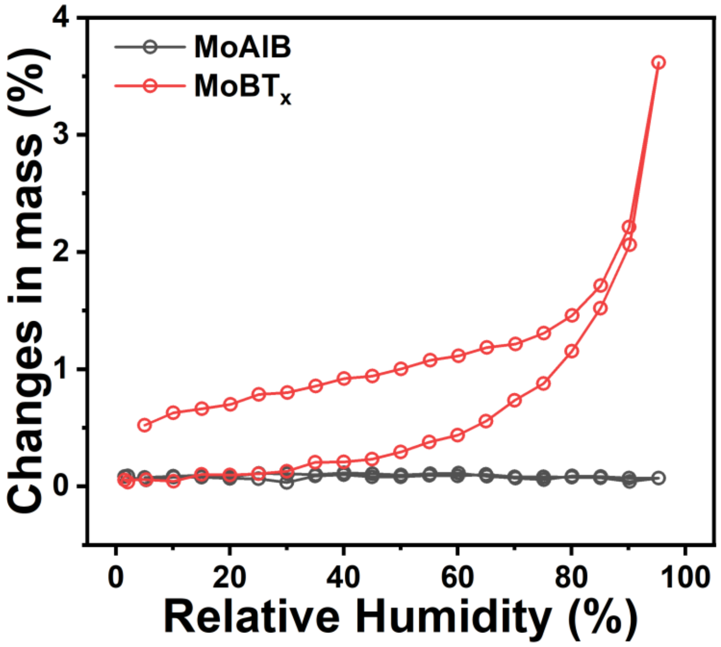

Figure S6. The dynamic vapour sorption (DVS) curve of MoAlB and $MoBT_x$ versus relative humidity at room temperature.

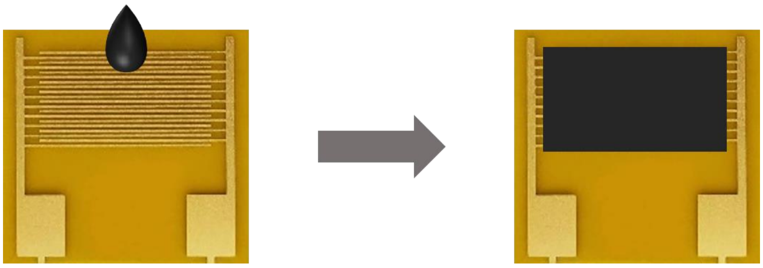

Figure S7. The schematic diagram of humidity sensor Fabrication.

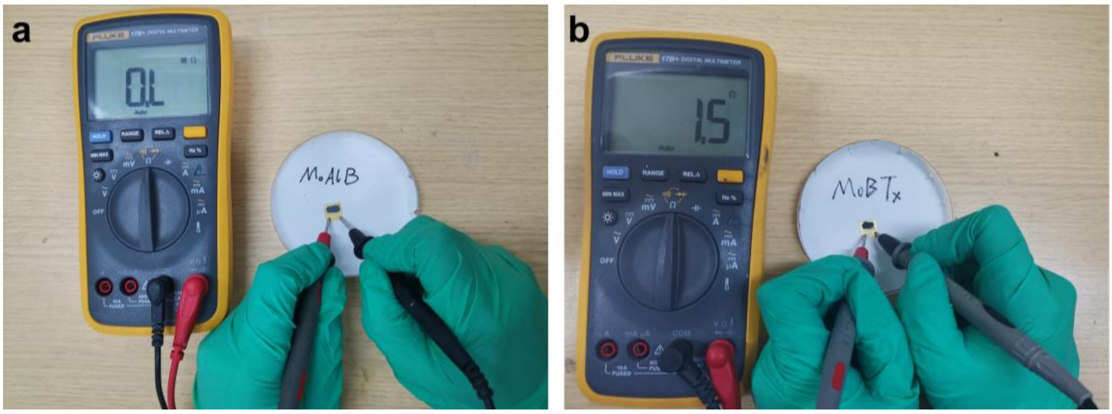

Figure S8. (a) High impedance state diagram of the MoAlB-based sensor. (b) Low resistance state diagram for the $MoBT_x$-based sensor.

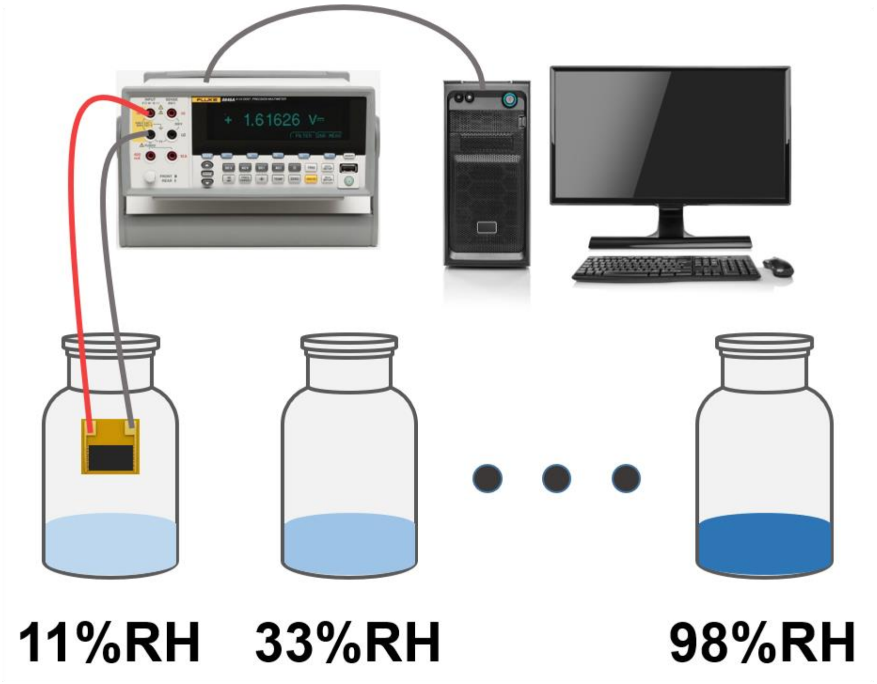

Figure S9. Static humidity test schematic diagram.

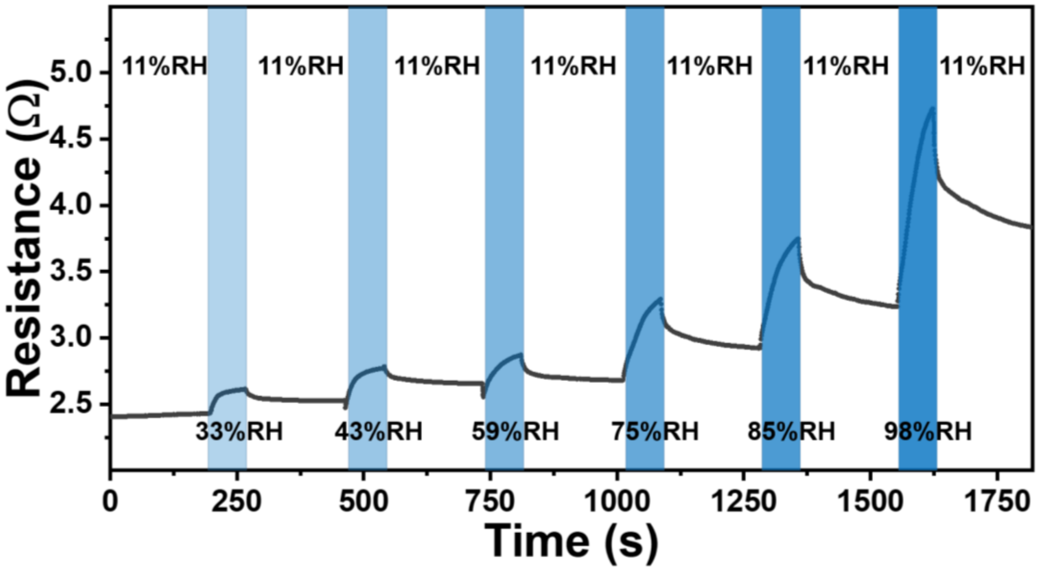

Figure S10. The response-recovery curves of the $MoBT_x$ sensor to different relative humidity at room temperature.

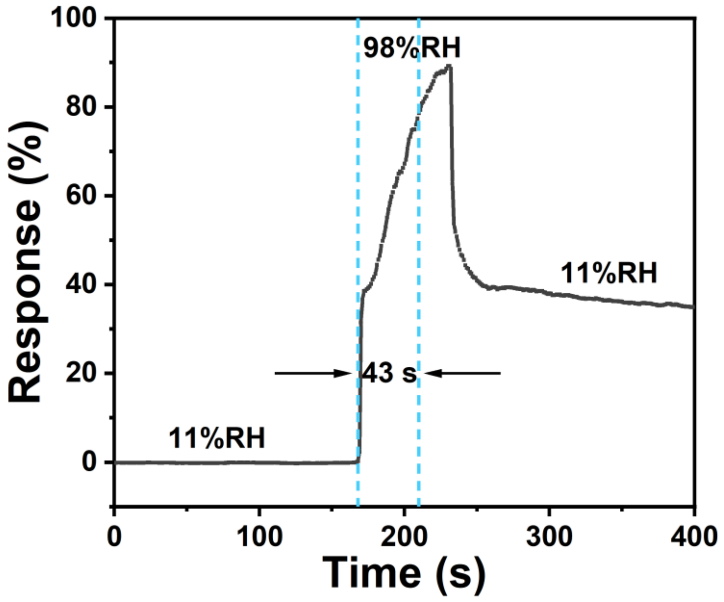

Figure S11. Response-recovery curve of the $MoBT_x$ sensor to 98%RH at room temperature.

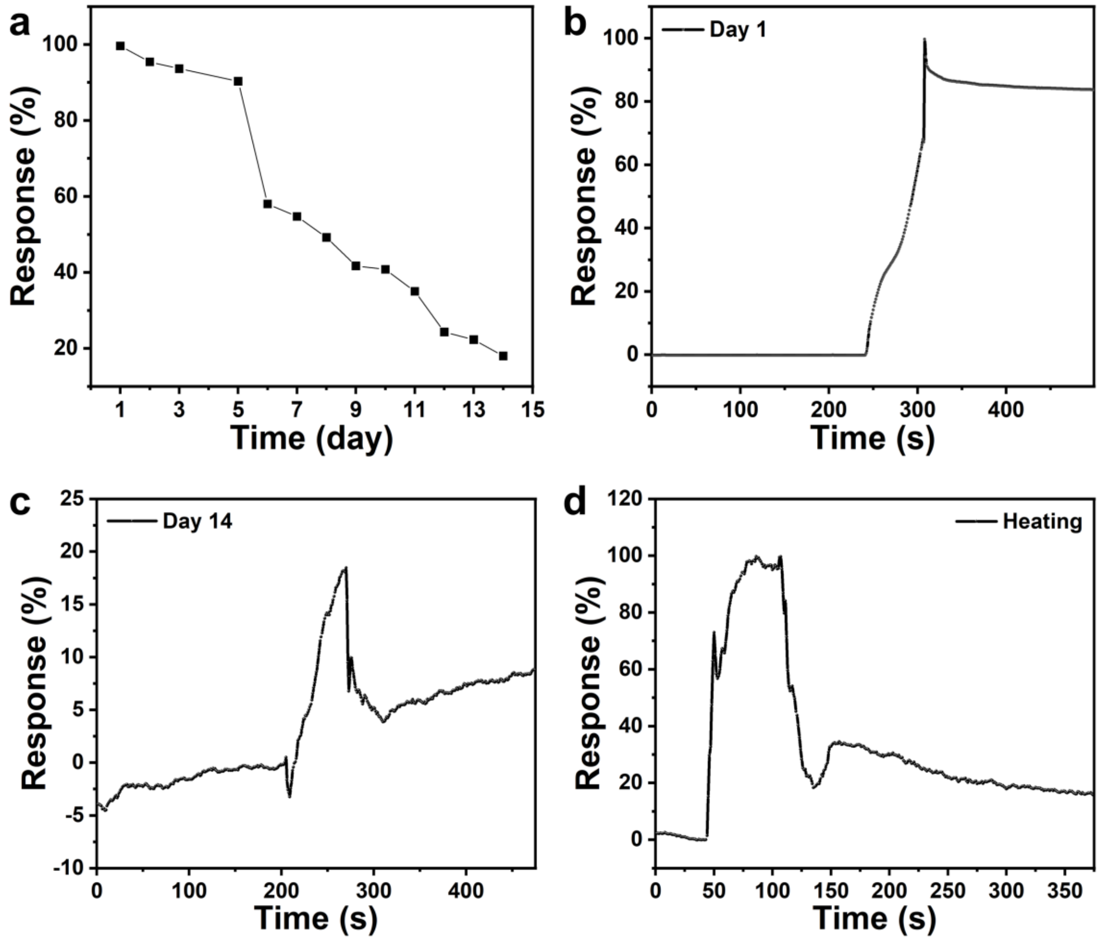

Figure S12. (a) Response values of the $MoBT_x$ sensor to 98% RH at room temperature within 14 days. Response-recovery curve of the $MoBT_x$ sensor to 98%RH at room temperature on (b) day 1 and (c) day 14. (d) Response-recovery curve of the $MoBT_x$ sensor to 98%RH with heating on day 15.

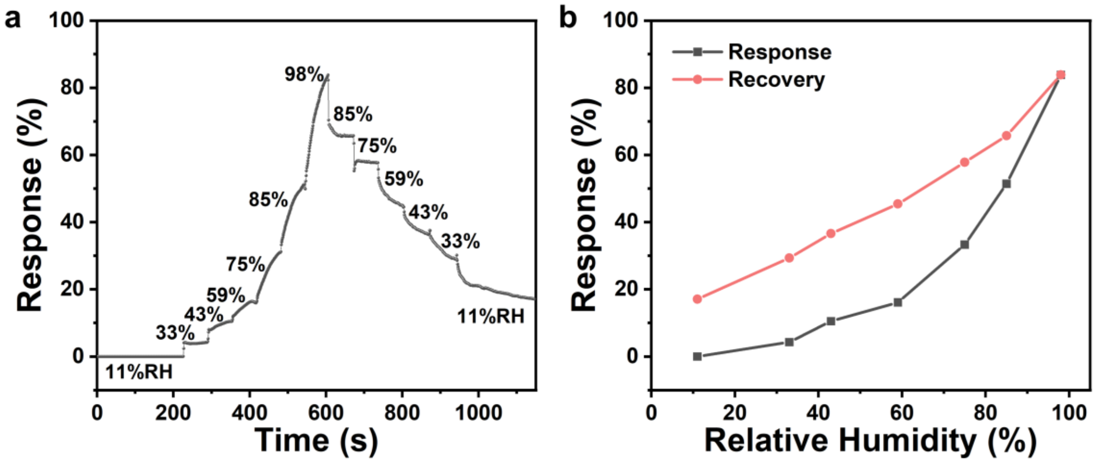

Figure S13. (a) Dynamic response and recovery characteristics of the $MoBT_x$ humidity sensor. (b) Hysteresis characteristics of the $MoBT_x$ humidity sensor in the range of 11-98% RH.

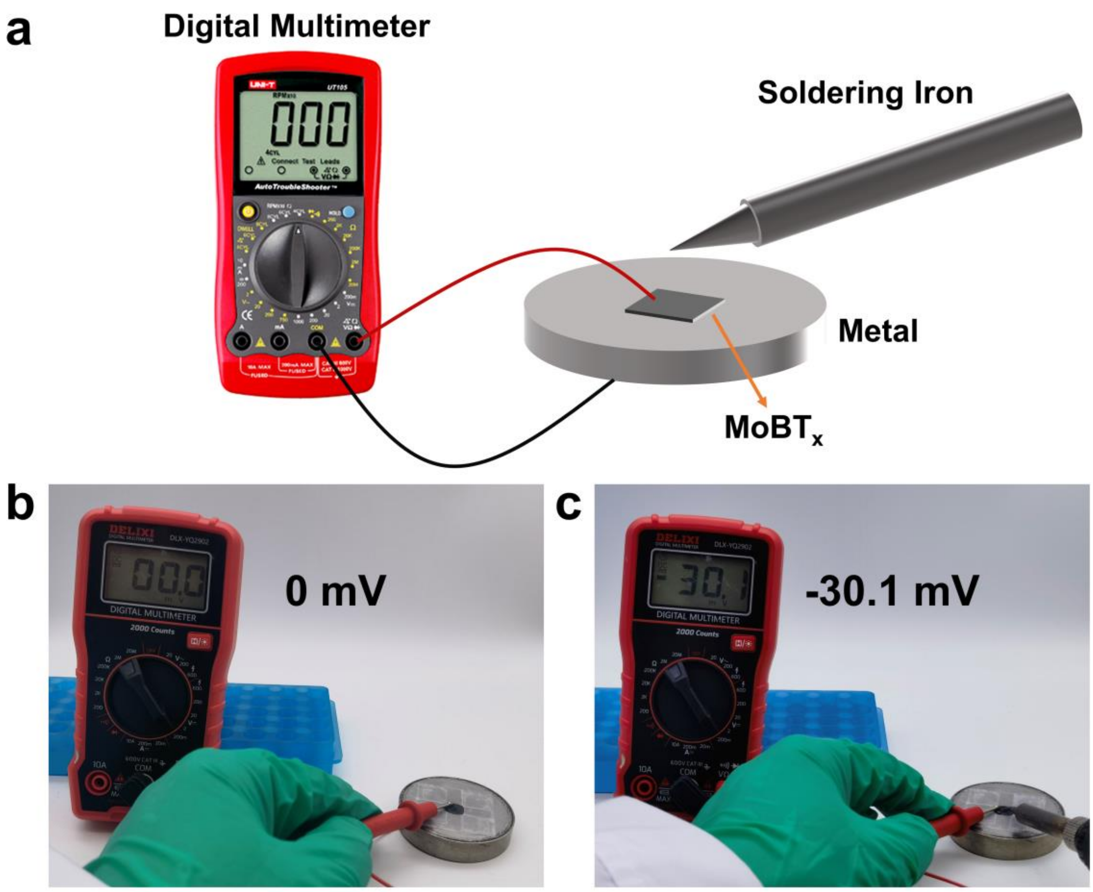

Figure S14. (a) Schematic diagram of thermoelectric effect test. (b) Zero voltage at initial state. (c) Negative voltage during heating state.

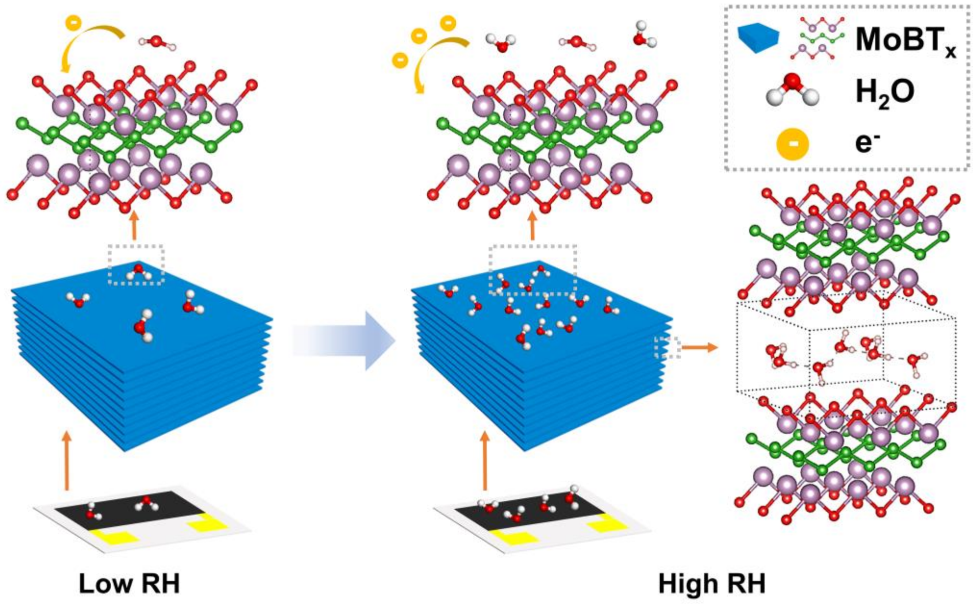

Figure S15. Schematic of the humidity-sensing mechanism of the $MoBT_x$ humidity sensors.

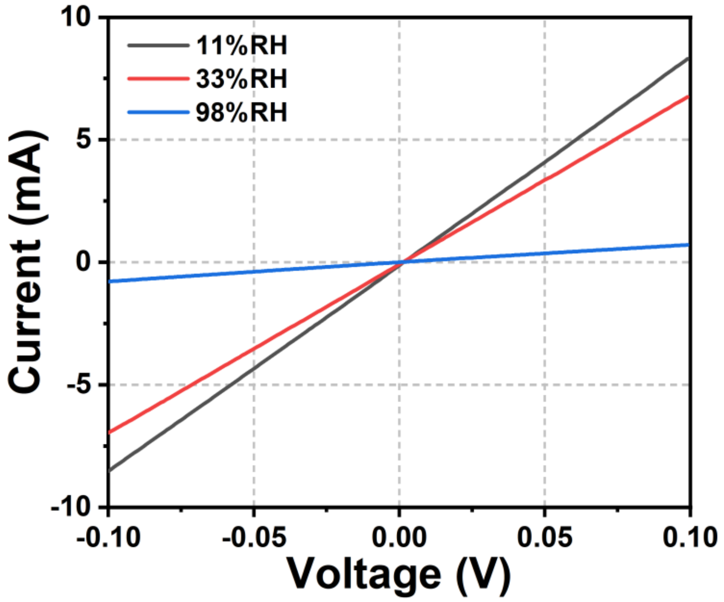

Figure S16. I-V test curve of the $MoBT_x$ sensor under 3 RH conditions at room temperature.

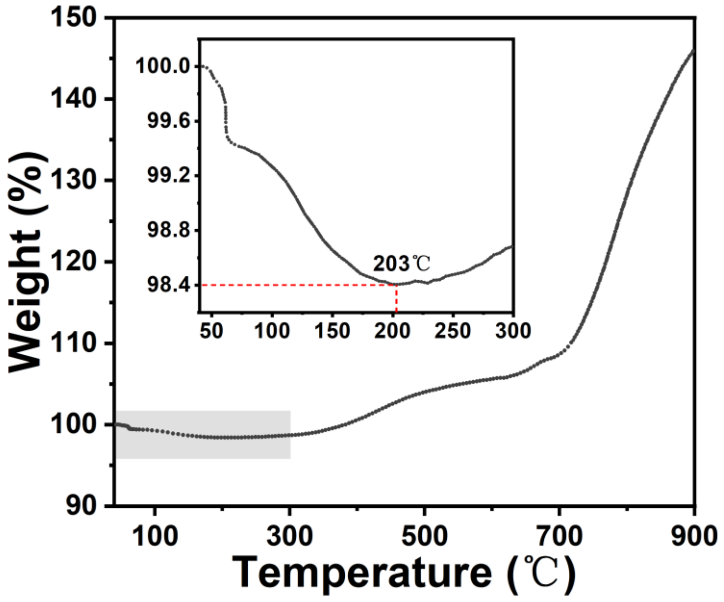

Figure S17. Thermogravimetric analysis thermograms of $MoBT_x$.

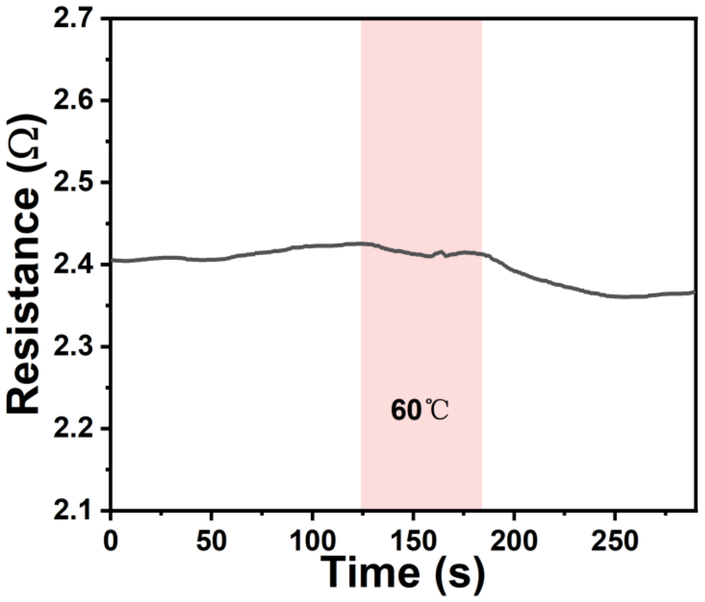

Figure S18. The resistance curve of the $MoBT_x$ sensor with short-time heating

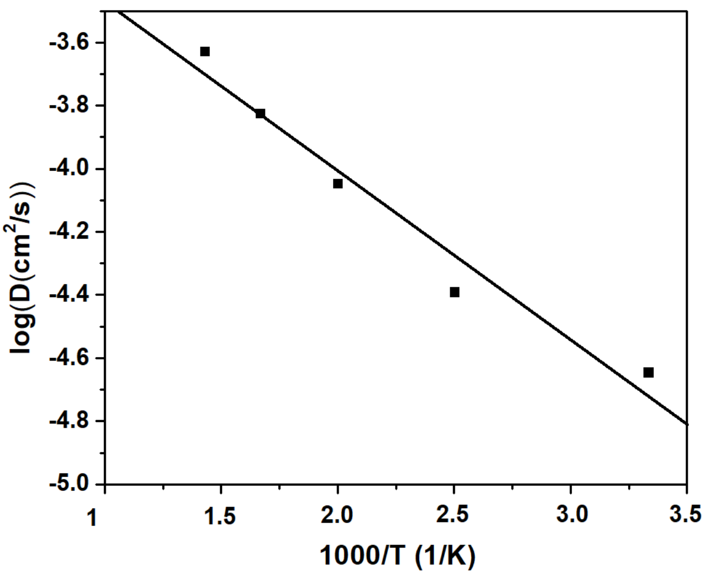

Figure S19. Arrhenius plot of the diffusion coefficient of water molecules on the MoBO surface.

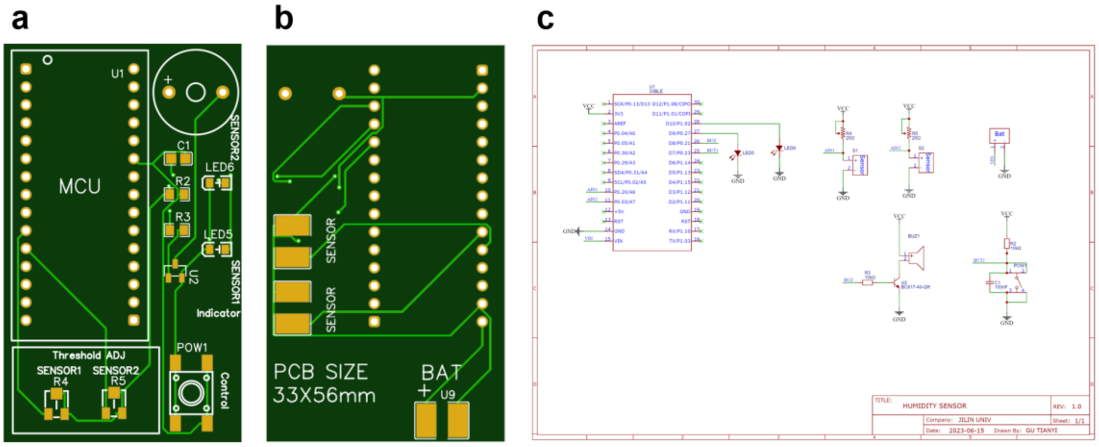

Figure S20. Humidity sensors warning circuit (a) front and (b) back, and (c) schematic circuit diagrams

Table S1. Testing of the sensor power consumption.

Resistance (Ω)	Voltage (mV)	Power Consumption (μW)
2.19	2.20	2.21
2.22	2.24	2.26
2.23	2.25	2.27

Table S2. The electrical properties of $MoBT_x$ film.

Magnetic (G)	Hall coefficient (cm^{-1} c^{-1})	Carrier density (cm^{-3})	Conductivity type	modifying factor
3000	0.8125	7.681×10^{18}	p	0.9723
6000	0.6535	9.550×10^{18}	p	0.9723

Table S3. Comparison of the recovery level (RL) of the sensor with and without heating during recovery at different relative humidity.

Relative Humidity	RL (Without Heating)	RL (Heating)
33%	46.94%	97.04%
43%	36.63%	92.21%
59%	43.66%	81.72%
75%	42.62%	88.19%
85%	38.73%	77.50%
98%	38.90%	82.10%

Table S4. The diffusion coefficient of water molecules on the MoBO surface at different temperatures.

Temperature (K)	Diffusion coefficient (cm^2/s) ^10^{-5}
300	2.27
400	4.07
500	9.00
600	15.00
700	23.60
